# Supplementary material for: Comprehensive data-driven analysis of the impact of chemoinformatic structure on the genome-wide biological response profiles of cancer cells to 1159 drugs
Source: BMC Bioinformatics. 2012 May 30;13:112. doi: 10.1186/1471-2105-13-112 (PMC3532323; doi:10.1186/1471-2105-13-112)

Figures

Publicly available human breast cancer data were obtained from the National Center for Biotechnology Information Gene Expression Omnibus (NCBI-GEO) microarray data repository. The NCBI-GEO (http://www.ncbi.nlm.nih.gov) is a repository for microarray gene expression and other molecular profile data generated from several research laboratories. It provides an online platform for gene expression data browsing, querying and retrieval of raw data files. Twelve HG-U133 Plus 2.0 affymetrix raw breast cancer series datasets were downloaded from GEO, series Accession (GSE7307, GSE13787, GSE3744, GSE12790, GSE6532, GSE10780, GSE5764, GSE9195, GSE5460, GSE13671, GSE8977 and GSE10810). In total we processed and normalized 179 normal and 500 cancer .CEL files using RMA-normalization method. Figure A, Figure B and Figure C correspond to this data, subset over genes from the specific component.

FIGURE A. Unsupervised Clustering of Human Breast Cancer Patients response profile over 3A-genes

Zoom to see details

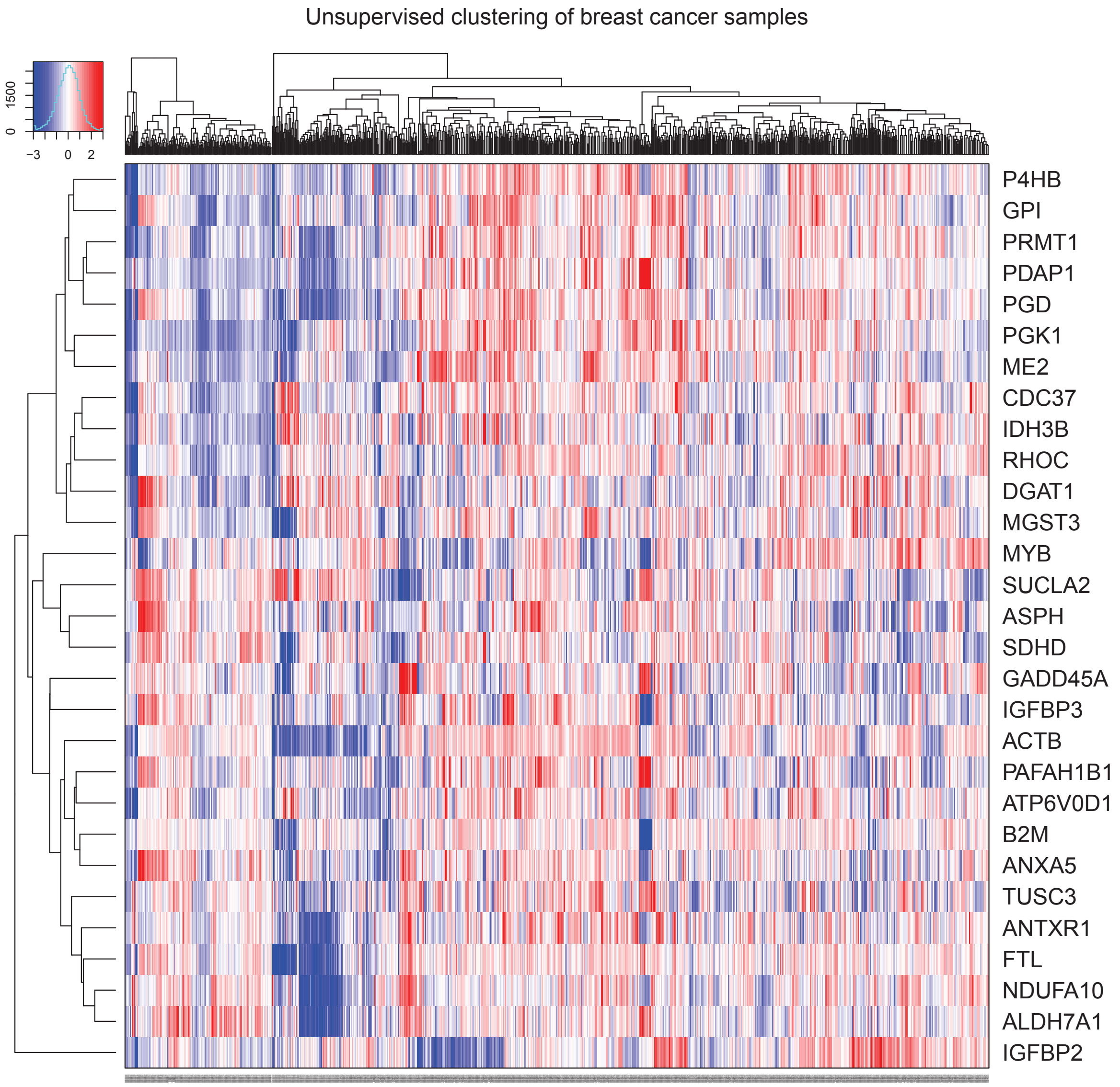

FIGURE B. Human Breast Cancer Patients response profile over 2B-genes

Zoom to see details

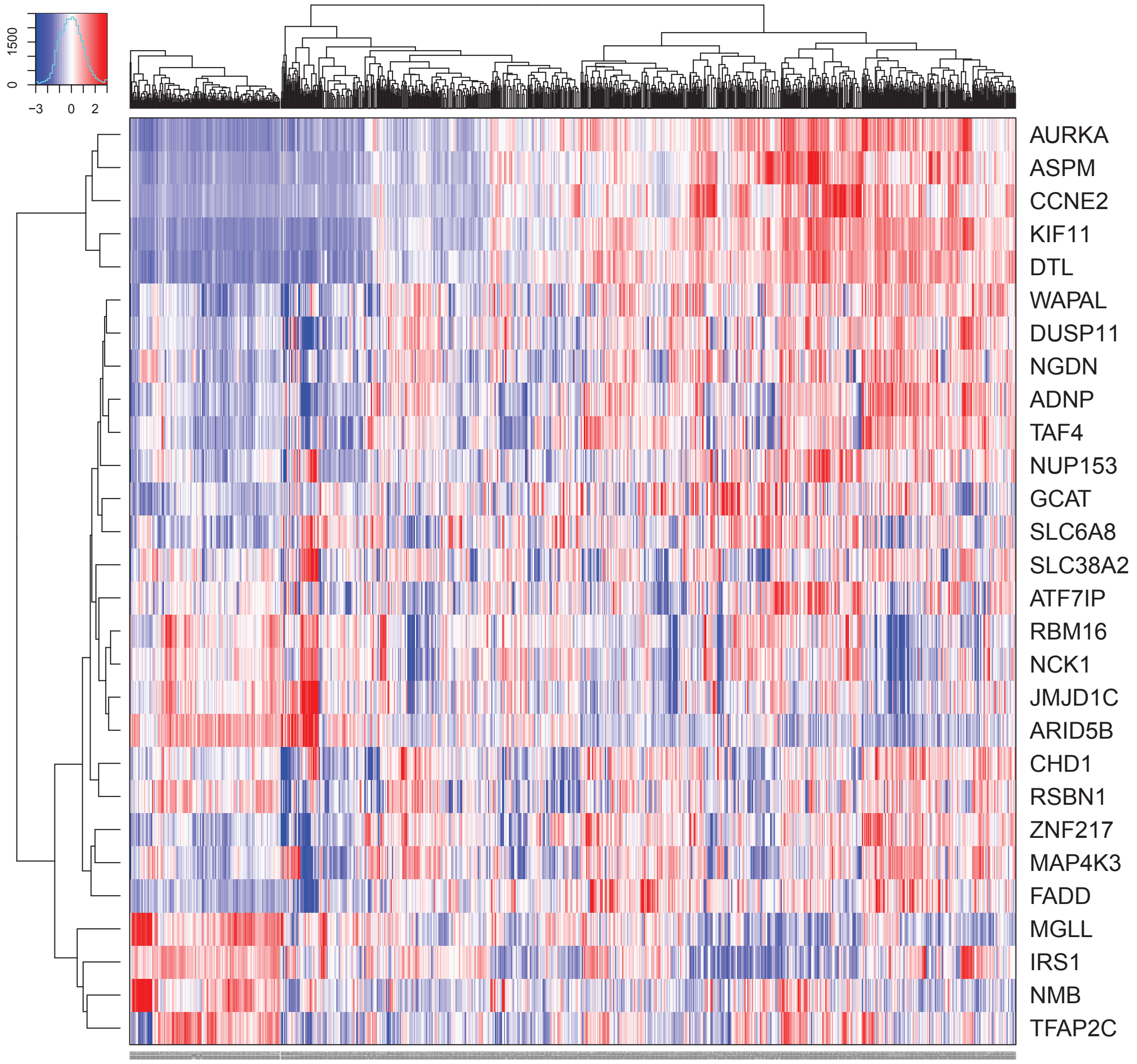

FIGURE C. Human Breast Cancer Patients response profile over 10A-genes

Zoom to see details

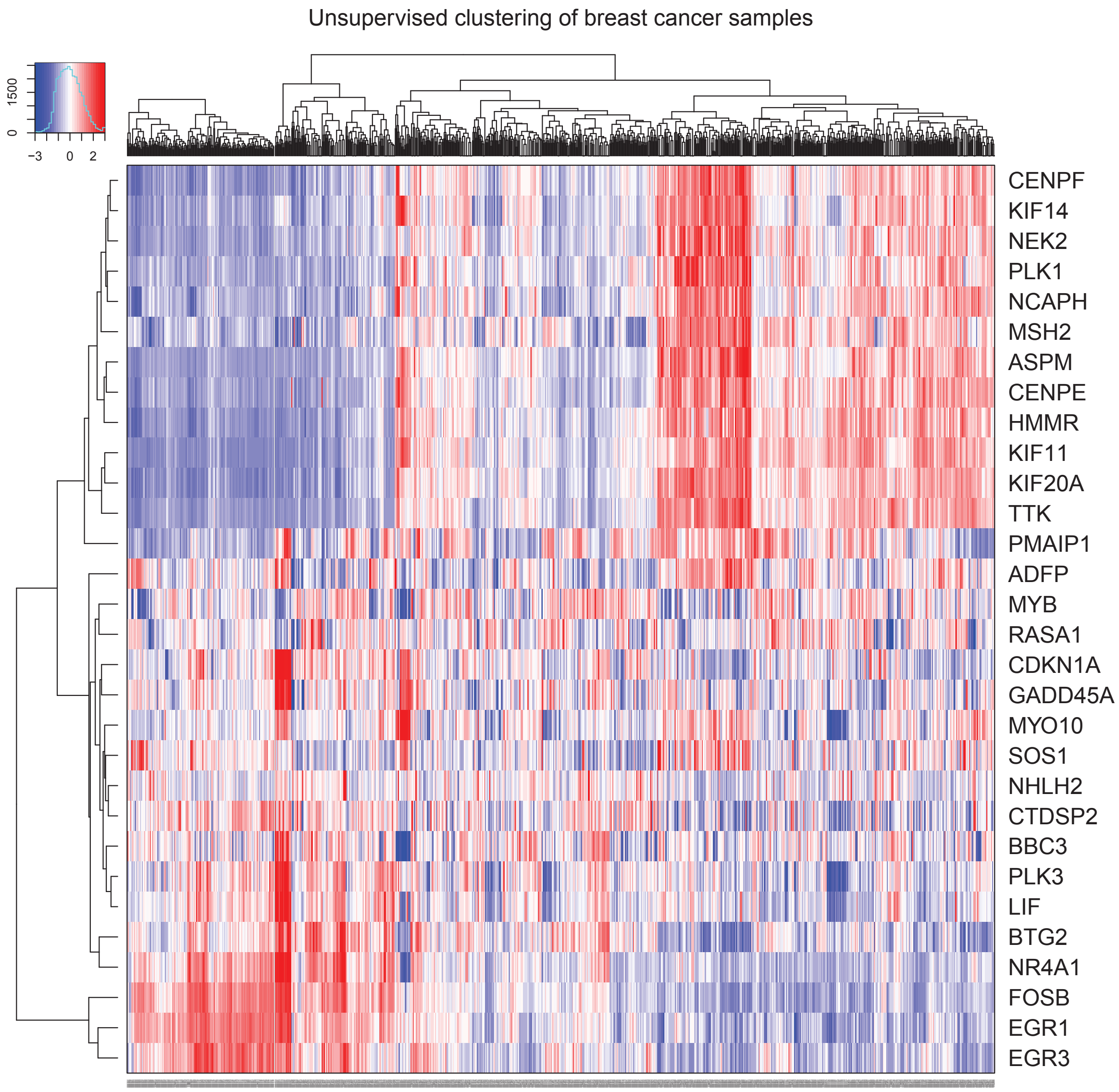

FIGURE D. Gene and Chemical Composition Similarity

Biological response and Chemical composition similarity across 20 subcomponents (represented as X and Y-axis). Colors depict the biological response similarity based on Jaccard similarity measure while the labels indicate the number of compounds (out of the top 20 for each component) shared between any two subcomponents.

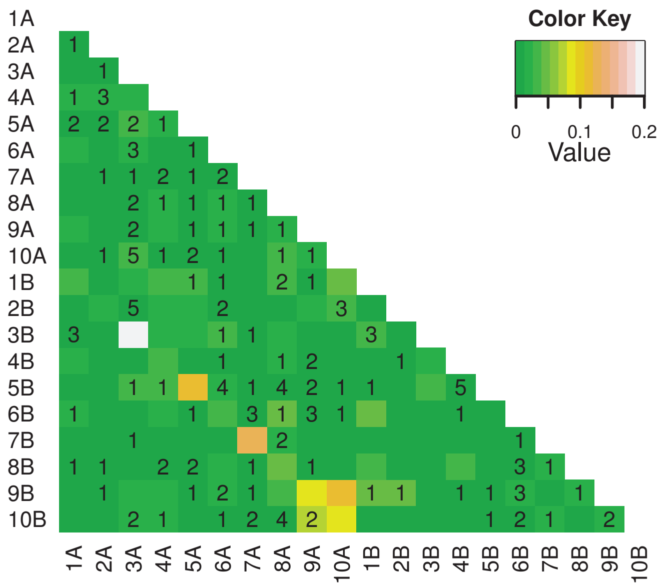

Supplement: Additional file 4 Figure S1. — HeatMaps.pdf. Contains breast cancer patient heat maps and chemical/biological composition plot. [file 1471-2105-13-112-S4.pdf]
